# Baricitinib: The First Jak Inhibitor Approved in Europe for the Treatment of Moderate to Severe Atopic Dermatitis in Adult Patients

**DOI:** 10.3390/healthcare9111575

**Published:** 2021-11-18

**Authors:** Giulia Radi, Oriana Simonetti, Giulio Rizzetto, Federico Diotallevi, Elisa Molinelli, Annamaria Offidani

**Affiliations:** Dermatological Clinic, Department of Clinical and Molecular Sciences, Polytechnic Marche University, Via Conca 71, 60020 Ancona, Italy; radigiu1@gmail.com (G.R.); grizzetto92@hotmail.com (G.R.); federico.diotallevi@hotmail.it (F.D.); molinelli.elisa@gmail.com (E.M.); a.offidani@ospedaliriuniti.marche.it (A.O.)

**Keywords:** atopic dermatitis, baricitinib, JAK inhibitors, efficacy, safety

## Abstract

**Background**: Atopic dermatitis (AD) is an inflammatory skin disease characterized by a wide phenotypic variety with a very complex pathophysiological mechanism that has led to the identification of new therapeutic targets, such as janus kinasis (JAK) inhibitors. **Objectives**: To evaluate the efficacy and safety of baricitinib, the first JAK 1 and 2 inhibitor approved in Europe for the treatment of adult patients with moderate-to-severe AD. **Methods**: The efficacy and safety data available from the Phase III studies belonging to the BREEZE AD program are presented. **Results:** Results from BREEZE-AD1, AD2, AD4, and AD7 showed the efficacy of Baricitib 4 mg, administered orally, once daily, as monotherapy or in combination with topical corticosteroid (TCS), with a significant proportion of patients achieving primary endpoints IGA 0–1 (16.4% vs. 4.8%; 13.8% vs. 4.5%; 21.7% vs. 9.7%; 30.6% vs. 14.7%) and EASI75 (24.8% vs. 8.8%; 21.1% vs. 6.1%; 31.5% vs. 17.2%; 47.7% vs. 22.9%) at week 16 (W16) compared to placebo, respectively. Baricitinib showed rapid improvement in symptoms, starting from week 1 of treatment at 4 mg dosage, with a good safety profile. Nasopharyngitis, upper respiratory tract infections (URIs), creatine phosphokinase (CPK) elevations, and headache were the most frequently reported adverse events. **Conclusions**: Following the efficacy and safety data on W 16 from the phase III BREEZE-AD studies, baricitinib has recently been approved in Europe for the treatment of moderate to severe AD in adult patients. Further data to evaluate long-term efficacy and safety in a real-life setting are needed.

## 1. Introduction

Atopic dermatitis (AD) is a chronic inflammatory skin disease characterized by itch and eczematous lesions. Considered in the past as an early childhood disease, with an estimated prevalence of 15–25%, recently, it has been suggested that AD is also very prevalent in adults, with rates ranging from 1 to 10% [1,2,3,4,5,6]. AD in adults may persist from childhood [7] or may begin or re-occur in adulthood; in fact, there are adult-onset variants, consisting about one-fourth of adult AD cases, that more frequently show atypical phenotypes with different localizations than the classic variants, such as head and neck, hand eczema, portrait dermatitis, and prurigo nodularis [8]. Behind the clinical variety of AD, there is a multifactorial etiology that combines genetic predisposing factors to environmental factors that together trigger a complex pathophysiological mechanism dictated by an imbalance in the response of T helper (Th) 1 and 2 lymphocytes, with a predominance of the Th2 response [9]. Understanding the inflammatory pathway that underlies atopic dermatitis has made it possible to discover new key molecules capable of providing useful therapeutic solutions with a molecular target. After successful results of dupilumab [10], a monoclonal antibody directed against α subunit of interleukine (IL)-13 e IL-4 receptor, several clinical trials are actually ongoing to approve new target molecules for the treatment of severe AD: monoclonal antibodies anti IL-13 [11,12] (tralokinumab and lebrikizumab), anti IL-31 [13] (nemolizumab), and small molecules, such as the janus kinasis (JAK) inhibitors [14] (abrocitinib, baricitinib, and upadacitinib). This is a brief, descriptive literature review that describes the mechanism of action of the JAK inhibitors and their potential role in the treatment of atopic dermatitis, with a particular attention to baricitinib. Finally, the efficacy and safety results currently available from the pivotal studies conducted on baricitinib are shown.

## 2. JAK Inhibitors: Mechanism of Action and Role in the Treatment of AD

The janus kinase–signal transducers and activators of transcription (JAK–STAT) pathway is essential for both immune and hematopoietic function, but it is also one of the key components in the pathogenesis of multiple immune-mediated conditions, including AD, rheumatoid arthritis, psoriatic arthritis, and inflammatory bowel disease. In fact, various pro-inflammatory cytokines are able to exert their pathophysiologic function through this intracellular signaling pathway [15]. The JAK family is composed of JAK1, JAK2, JAK3, or tyrosine kinase 2 (TYK2) [16]. Once activated, JAKs phosphorylate the intracellular cytokine receptor domain, resulting in a docking site for STATs [17]. The overall result of JAK-STAT activation is the contribution to both AD skin inflammation and chronic itch by the promotion of the pro-inflammatory signaling, involving keratinocytes, neuronal cells, and innate and acquired immunity cells. Several molecules are employed in cutaneous immunoregulation: Interferon (IFN)gamma; type 2 cytokines, including IL-4 and IL-13, IL-31; and thymic stromal lymphopoietin (TSLP). After binding their own receptor, these chemokines activate JAK-STAT intracellular pathway, mainly promoting the phosphorylation of JAK1/JAK3, and STAT3, STAT5, and STAT6 signaling. The target cells are multiple: in the keratinocytes, JAK-STAT activation promotes cellular differentiation process and keratinocyte-derived chemokine and antimicrobial peptide production that make keratinocytes active components of the inflammatory process, leading to the skin barrier dysfunction. JAK-STAT is also the intracellular signaling in Th2 cells that sustain the inflammatory loop by the activation and differentiation of the same Th2 and the innate immune cells and by the synthesis of pro-inflammatory cytokines IL-4 and IL-13, directed to keratinocytes, eosinophils, fibroblasts, and B lymphocytes. Moreover, the JAK-STAT pathway plays a crucial role in the genesis of atopic pruritus through multiple effectors: in addition to promoting the synthesis of the main pruritogenic cytokine IL-31 in Th2, it is activated by binding to IL-4ra and IL-31 receptors in sensory neurons (JAK1/JAK2 and STAT 5) and by binding to transient receptors of vanilloid potential 1 (TRPV1) in dorsal root ganglia [18,19,20]. JAK inhibitors target the various kinases, causing suppression in the activity of one or more of these targets [20]. It is essential for granulocyte colony-stimulating factor, which stimulates the production of granulocytes and stem cells and interferons [21]. Moreover, JAK activation induces cell migration, differentiation, cell proliferation, and apoptosis. Thus, the therapeutic inhibition of JAK–STAT proteins by small-molecule inhibitors modulates immune cell function by detaching cells from cytokine with an anti-inflammatory, immunosuppressive, and antiproliferative effect [22]. Finally, given the multiple implications in the genesis of pruritus of the JAK-STAT pathway, JAK inhibitors also act specifically to reduce the itch. A growing body of literature has demonstrated that JAK inhibitors are safe and effective in multiple inflammatory skin conditions, extending the investigation of JAK inhibitors as a treatment for AD [23]. Among JAK inhibitor drugs currently employed in the treatment of AD, three molecules are under study on phase III clinical trials: upadacitinib and abrocitinib, belonging to the second generation of JAK inhibitors for their single specific inhibition of JAK1, and baricitinib, a selective and reversible inhibitor of JAK 1 and JAK2. Due to its blocking of multiple JAKs, baricitinib, together with tofacitinib and ruxolitinib, are employed in the treatment of immune-mediated diseases different from AD and belong to first generation of JAK inhibitors. In detail, baricitinib has been recently approved by EMA (European Medicine Agency) for the treatment of AD: on 17 September 2020, the Committee for Medicinal Products for Human Use (CHMP) adopted the new indication for AD as follows: baricitinib is indicated for the treatment of moderate to severe atopic dermatitis in adult patients who are candidates for systemic therapy [24].

## 3. Efficacy and Safety Results from Phase II and III Trials

### 3.1. Methods

A search was conducted using PubMed/MEDLINE, Embase, Cochrane Skin databases, and Clinicaltrials.gov with the search terms “atopic dermatitis” or “atopic eczema” and “baricitinib”. The aim of this review was the evaluation of the available safety and efficacy results of baricitinib for the treatment of AD from randomized controlled trials (RCTs) of phase II and trials belonging to phase III BREEZE-AD program. To facilitate understanding and reading, study protocols, summaries of results and data sources are given in tables and graphs. Finally, the impact on therapeutic and economic management for the treatment of atopic dermatitis, following the approval of the new molecule, is discussed.

Eight RCTs are part of the study protocol of baricitinib in the treatment of moderate to severe atopic dermatitis in adult patients. The phase II trial has been concluded with the enrollment of 124 patients, and the first results were published in March 2018 [25]. The BREEZE-AD program includes seven phase III studies [26,27,28,29,30,31,32] evaluating the efficacy and safety of baricitinib in the treatment of moderate to severe atopic dermatitis in adult patients. In addition, a further phase III study in adolescents is underway [33]. Among the phase III studies of the BREEZE-AD program, two were limited to the USA and Canada, and five of them involved several global countries for a total of 4647 participants over the age of 18 years. With the exception of AD6, which is an open-label study of baricitinib 2 mg, all other studies are double-blind randomized controlled trials in which treatment arms at different therapeutic doses of baricitinib are compared with placebo. Pointedly, in AD1 and AD2, baricitinib was administered in monotherapy, while in AD3 and AD5, topical corticosteroids (TCS) were allowed; in AD4 and AD7, the TCS were combined to oral baricitinib administration as for protocol. Study duration, primary end-point, different dosage evaluated, and other main characteristics of the BREEZE AD program phase III studies are summarized in Table 1. Efficacy results from BREEZE AD 3 and AD6 are not currently available on clinicaltrials.gov. Several articles have been published in the literature on efficacy and safety data obtained from the results of RCTs: the results are described below and summarized in Table 2.

### 3.2. Efficacy

Consistent efficacy data about baricitinib for the treatment of severe AD in adult patients are currently available from phase III studies at W16. In detail, BREEZE-AD1, AD2, AD5, and AD7 trials were recently concluded, and further data are also available from preliminary results of AD4.

#### 3.2.1. Phase II Trials

Encouraging results from the phase II randomized, double-blinded, placebo-controlled study have been reported about the efficacy of baricitinib in combination with topical corticosteroids in adult patients with moderate to severe AD at W16 [34]. One hundred twenty-four subjects were successfully enrolled and randomized in a 4:3:3 ratio, corresponding to QD (quaque die) placebo or 2 mg or 4 mg baricitinib. The primary endpoint was the proportion of participants achieving 50% or greater improvement in the baseline Eczema Area Severity Index (EASI) score at week 16 (EASI50 at week 16). Patients who received 4 mg baricitinib achieved a significant improvement of at least 50% in EASI compared to patients who had received placebo (61% vs. 37%, respectively) at 16 weeks [34].

#### 3.2.2. Phase III Trials

##### IGA 0–1 and EASI75 Results

The efficacy of baricitinib 4 mg administered in monotherapy has been confirmed in both phase III trials BREEZE-AD1 and AD2 [35]. In total, 624 patients were enrolled in BREEZE-AD1, and 615 patients were enrolled in BREEZE-AD2. The primary endpoint Investigator global assessment (IGA) 0–1 was achieved by a significantly higher proportion of patients in the 4-mg and also in the 2-mg group compared to placebo (respectively, 16.4% (*p* < 0.001), 11.4% (*p* < 0.05), and 4.8% for AD1 and 13.8% (*p* = 0.001), 10.6% (*p* < 0.05), and 4.5% for AD2). Baricitinib 4 mg provided a significant improvement in EASI score at W16, with a 59.4% and 54.9% reduction in BREEZE-AD1 and BREEZE-AD2, respectively. The realization of the other secondary efficacy endpoints (EASI75, EASI90, and SCOring AD (SCORAD) 75 at week 16) was detected in a significantly higher proportion of participants in the 4-mg groups (*p* = 0.001). Efficacy results shown in clinical trials BREEZE-AD 7 and AD4 confirmed the effectiveness of baricitinib in the treatment of moderate to severe AD in adults patients even in association with the administration of low- and medium-potency TCS. In BREEZE-AD7, among 329 patients enrolled, at W16, the proportion of patients who reached the primary end point of vIGA-AD score of 0 or 1 was significantly higher for patients treated with 4 mg of baricitinib vs. placebo (31% vs. 14%; *p* = 0.004), while the primary end point for 2 mg of baricitinib was not met (24%; *p* = 0.08). The proportion of patients who gained an EASI75 response at week 16 was significant in both 4-mg and 2-mg groups (48 and 43%, respectively) compared to placebo group (23%) [36]. In BREEZE-AD4, the primary endpoint was the percentage of participants achieving an EASI75 response at W16 from baseline. The results showed a significant difference in the proportion of patients who reached the EASI75 in 4 mg group (31.5%; *p* ≤ 0.05) but not in 2 mg group (27.6%; *p* = 0.07) compared to placebo (17.2%). Similarly, the main secondary endpoint (IGA 0 or 1) was achieved by a proportion of patients statistically significant only for 4-mg group (21.7%; *p* ≤ 0.05) rather than 2-mg group (15.1%; *p*= 0.2) compared to placebo (9.7%) [29].

**Figure 1:**
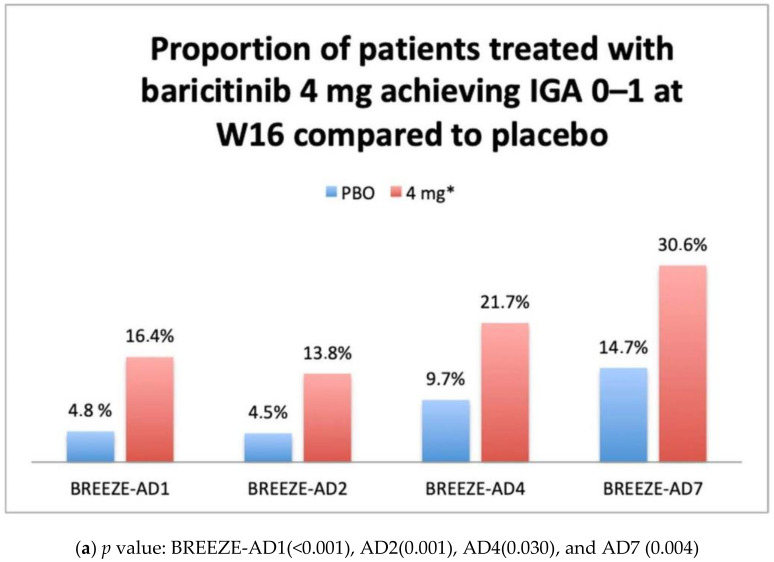

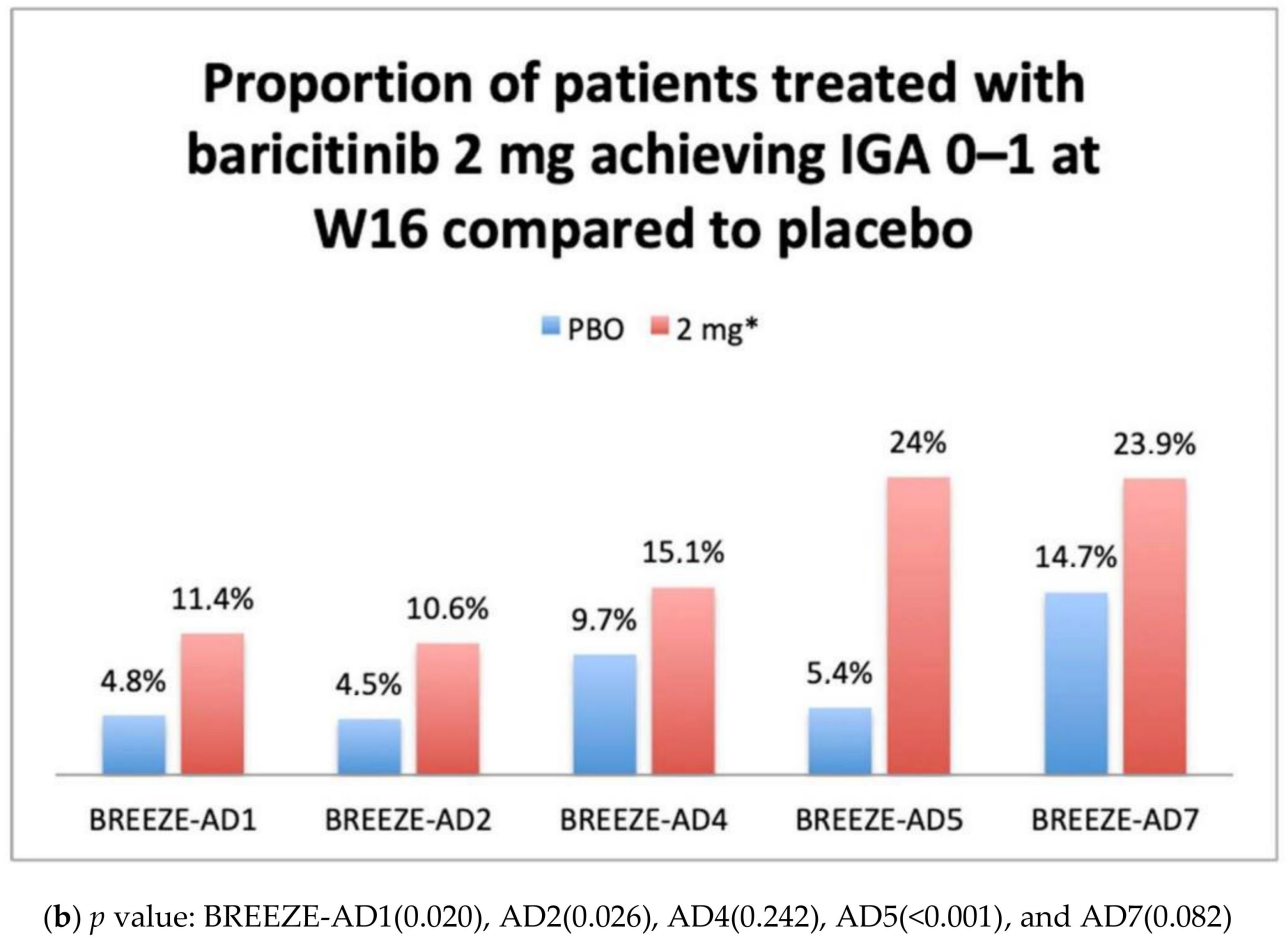
(**a**) Primary efficacy endpoint from BREEZE-AD1, AD2, AD4, and AD7: Proportion of patients treated with baricitinib 4 mg achieving IGA 0–1 at W16 compared to placebo. Abbreviation: PBO, placebo; IGA, investigator global assessment; W16, week 16; Bari, baricitinib: * In BREEZE-AD-4 and AD7, baricitinib was administered in association with topical corticosteroid as for protocol. (**b**) Primary efficacy endpoint from BREEZE-AD1, AD2, AD4, AD5, and AD7: Proportion of patients treated with baricitinib 2 mg achieving IGA 0–1 at W16 compared to placebo. Abbreviation: PBO, placebo; IGA, investigator global assessment; W16, week 16; Bari, baricitinib. * In BREEZE-AD 4 and AD7, baricitinib was administered in association with topical corticosteroid as for protocol.

**Figure 2:**
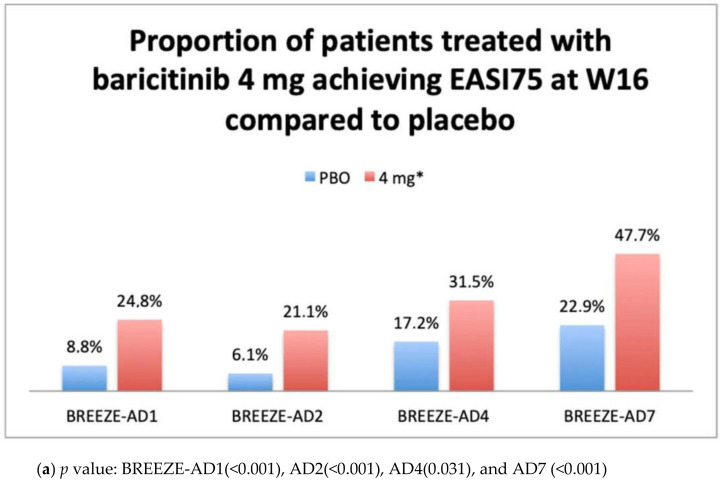

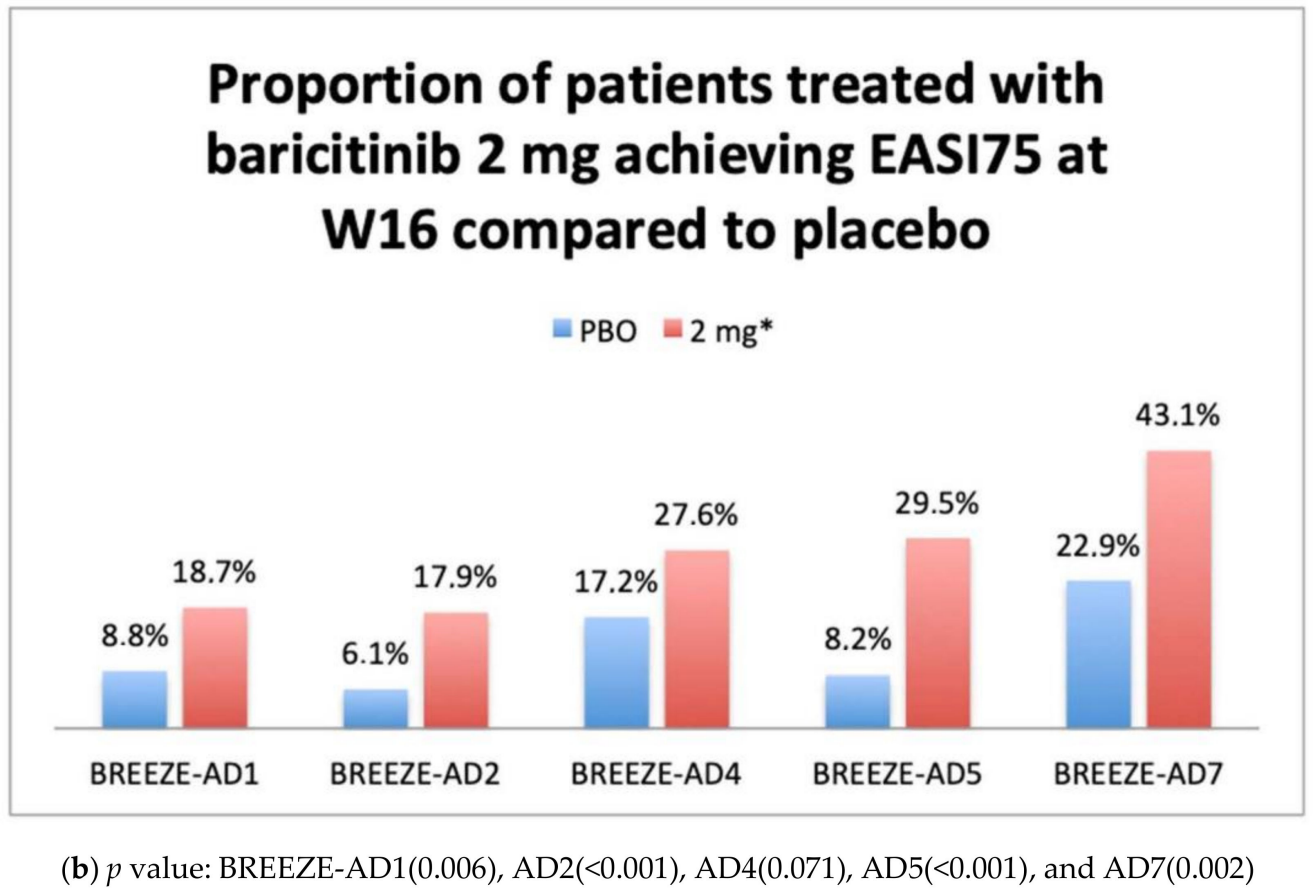
(**a**) Primary efficacy endpoint from BREEZE AD1, AD2, AD4, and AD7: Proportions of patient treated with baricitinib 4 mg achieving EASI75 at W16 compared to placebo. Abbreviations: PBO, placebo; EASI, Eczema Area Severity Index; W16, week 16. * In BREEZE-AD 4 and AD7, baricitinib was administered in association with topical corticosteroid as for protocol. (**b**) Primary efficacy endpoint from BREEZE-AD1, AD2, AD4, AD5, and AD7: Proportions of patient treated with baricitinib 2 mg achieving EASI75 at W16 compared to placebo. Abbreviations: PBO, placebo; EASI, Eczema Area Severity Index; W16, week 16. * In BREEZE-AD 4 and AD7, baricitinib was administered in association with topical corticosteroid as for protocol.

**Figure 3:**
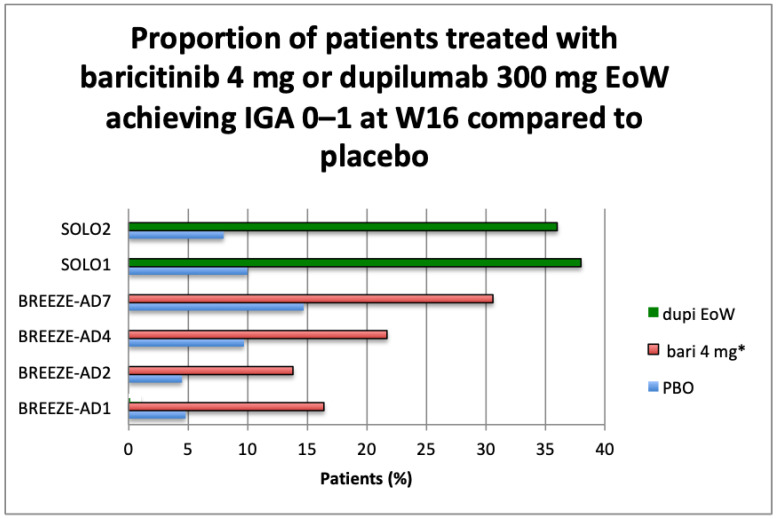
Overview of primary efficacy endpoint from RCTs: Proportion of patients treated with baricitinib 4 mg or dupilumab 300 mg EoW achieving IGA 0–1 at W16 compared to placebo. Abbreviations: PBO, placebo; IGA, investigator global assessment; W16, week 16; EoW, every other week; Dupi, dupilumab; bari, baricitinib. * In BREEZE-AD 4 and AD7, baricitinib was administered in association with topical corticosteroid as for protocol.

**Figure 4:**
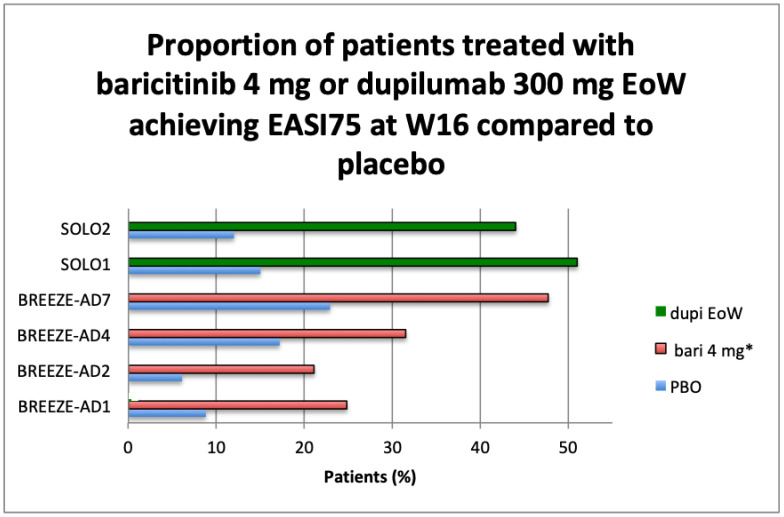
Overview of primary efficacy endpoint from RCTs: Proportion of patients treated with baricitinib 4 mg or dupilumab 300 mg EoW achieving EASI75 at W16 compared to placebo. Abbreviations: PBO, placebo; IGA, investigator global assessment; W16, week 16; EoW, every other week; dupi, dupilumab; bari, baricitinib. * In BREEZE-AD 4 and AD7, baricitinib was administered in association with topical corticosteroid as for protocol.

BREEZE-AD5 was limited to U.S. and Canada populations and evaluated the efficacy of baricitinib 1 mg or 2 mg versus placebo administered QD orally in monotherapy in adults suffering from moderate to severe AD. At week 16, the proportion of patients achieving the primary endpoint EASI75 was 8, 13, and 30% (*p* < 0.001, 2 mg vs. placebo), and those with a vIGA 0–1 were 5, 13, and 24% (*p* < 0.001, 2 mg vs. placebo) for placebo, baricitinib 1 mg, and baricitinib 2 mg, respectively [37]. Main primary outcomes results available from clinicaltrials.gov are summarized in graphs (Figure 1a,b, Figure 2a,b, Figure 3 and Figure 4).

##### Patient-Reported Outcomes PROs

**Figure 5:**
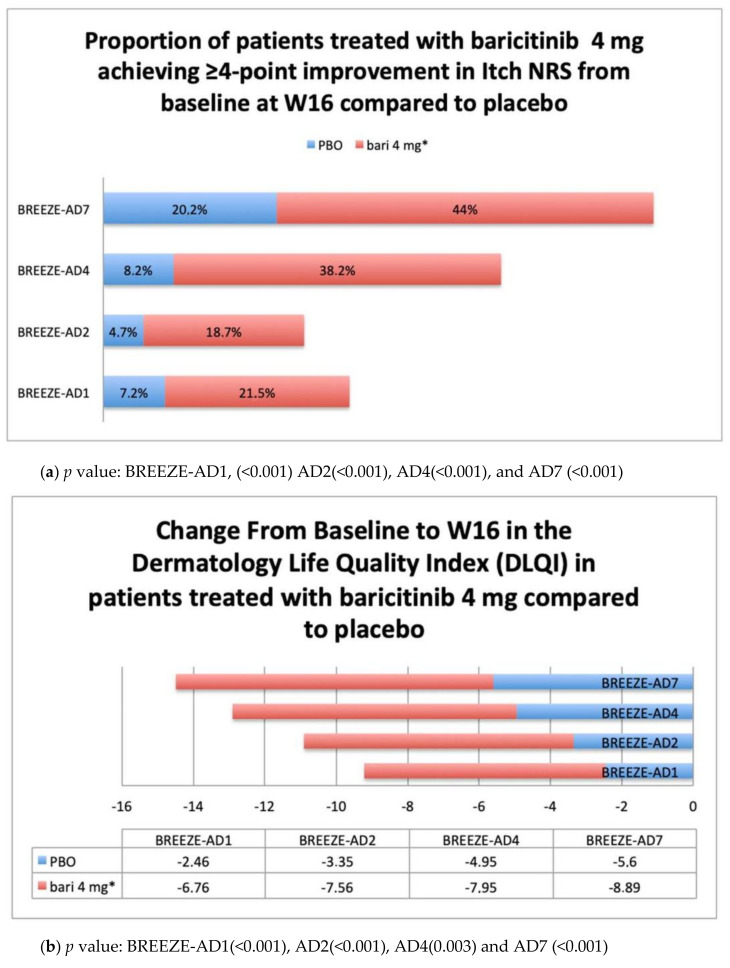

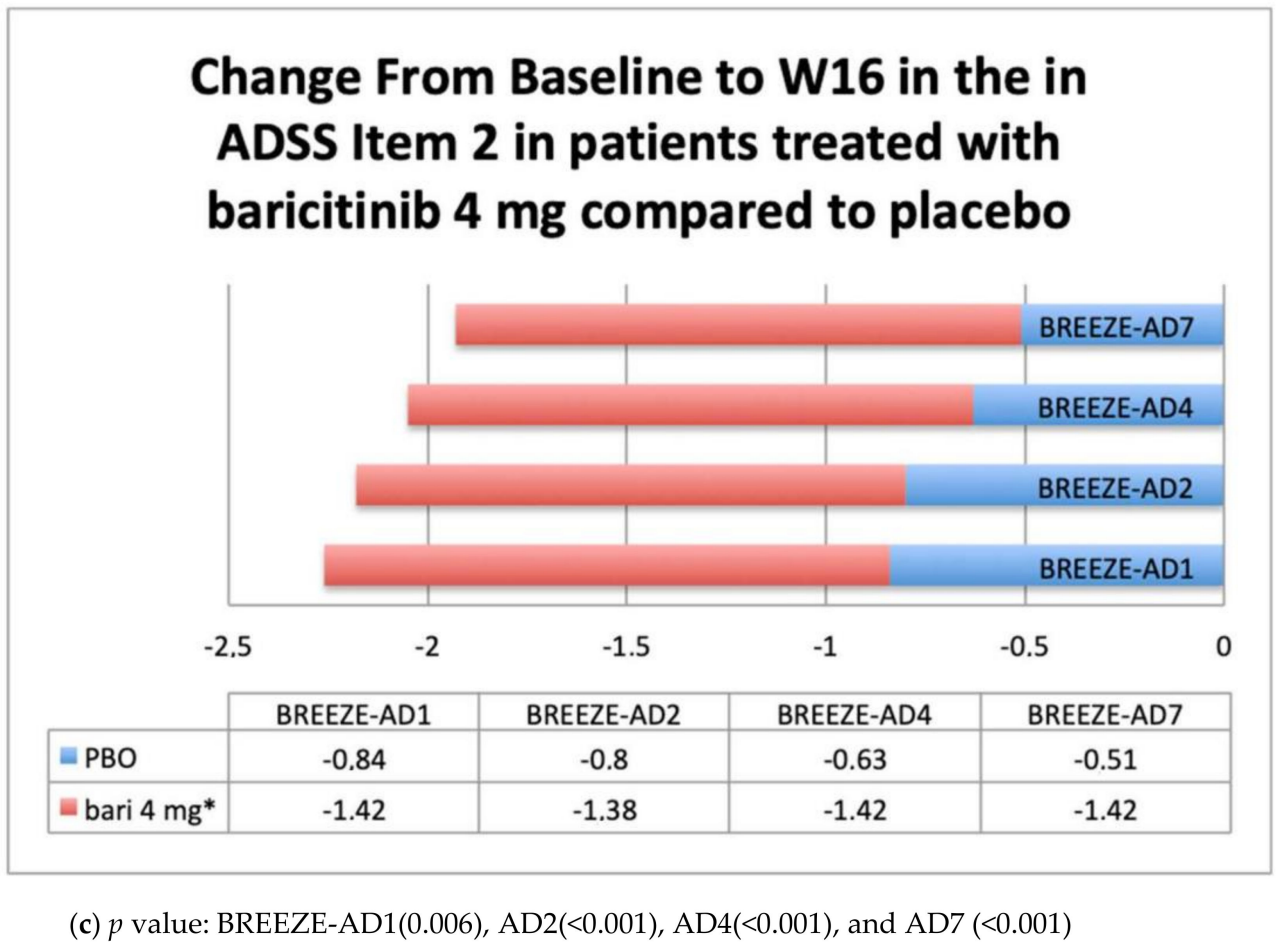
(**a**) PROs endpoints from the BREEZE-AD1, AD2, AD4, and AD7: Proportion of patients treated with baricitinib 4 mg achieving ≥4-point improvement in Itch NRS from baseline at W16 compared to placebo. Abbreviations: PBO, placebo; NRS, Numeric Rating Scale; W16, week 16; bari, baricitinib. * In BREEZE-AD4 and AD7, baricitinib was administered in association with topical corticosteroid as for protocol. (**b**) PROs endpoints from the BREEZE-AD1, AD2, AD4, and AD7: Change from Baseline to W16 in the Dermatology Life Quality Index (DLQI) in patients treated with baricitinib 4 mg compared to placebo. Abbreviations: PBO, placebo; DLQI, Dermatology Life Quality Index; W16, week 16; bari, baricitinib. * In BREEZE-AD4 and AD7, baricitinib was administered in association with topical corticosteroid as for protocol. (**c**) PROs endpoints from the BREEZE-AD1, AD2, AD4, and AD7: Change from Baseline to W16 in the in ADSS Item 2 in patients treated with baricitinib 4 mg compared to placebo. Abbreviations: PBO, placebo; ADSS, Atopic Dermatitis Symptom Score; W16, week 16; bari, baricitinib. * In BREEZE-AD 4 and AD7, baricitinib was administered in association with topical corticosteroid as for protocol.

The Patient-Reported Outcomes (PROs) have been performed weekly in all cited phase III trials, starting from the first week of treatment to the end of the period of observation (W16). Main PROs results available from clinicaltrials.gov are summarized in graphs (Figure 5a–c) In BREEZE-AD1 and AD2, the improvement in itch was realized as early as week 1 for 4 mg and week 2 for 2 mg, while improvements in night-time awakenings, skin pain, and quality-of-life measures were observed by week 1 for both 4 mg and 2 mg (*p* ≤ 0.05) [35]. Reich et al. analyzed the results of the patient-reported outcomes from trials BREEZE-AD 1 and AD2 and they observed at W16, a significant reduction of itch severity in terms of Itch Numeric Rating Scale (NRS), respectively: in BREEZE-AD1: percent change from baseline −36.6% (4 mg) and −29.4% (2 mg) vs. placebo (−12.0%), *p* ≤ 0.001 and *p* ≤ 0.05; BREEZE-AD2: −47.2% (4 mg) and −46.9% (2 mg) vs. placebo (−16.6%), *p* ≤ 0.001). Improvements in SCORAD pruritus, Patient Oriented Eczema Measure (POEM) itch, skin pain severity, and sleep disturbance for both baricitinib treatment arms were also observed [38]. As regards BREEZE-AD7, statistically significant recovery was observed in Dermatology Quality of Life Index (DLQI) ≥4-point improvement, starting at week 2 in 4-mg plus TCS and 2-mg plus TCS groups (*p* ≤ 0.001 and *p* ≤ 0.05, respectively), in (Work Productivity and Activity Impairment) WPAI-AD at Week 1 in 4 mg plus TCS and 2 mg plus TCS groups (*p* ≤ 0.01; *p* ≤ 0.05, respectively) and in Patient Reported Outcomes Measurement Information System) PROMIS itch interference at Week 2 in 4-mg plus TCS group (*p* ≤ 0.01). [39].

Moreover, Wollemberg et al., by analyzing the results of BREEZE-AD7, focused on the impact of baricitinib + TCS on patient-reported measures of health-related quality of life (HRQoL), AD symptom impact, work and daily life functioning, and treatment benefit. The authors reported statistically significant improvements in itching and its impact on quality of life, sleep, and work activity in subjects treated with 4 mg + TCS compared with placebo + TCS starting at W1 and W2 of treatment. More delayed statistically significative improvements in patients treated with baricitinb 2 mg + TCS were observed starting from W4. Both treatment arms resulted in early benefits in HRQoL, symptom impact, and patient function across life domains that were sustained to week 16 and resulted in overall treatment benefit [39].

A recent analysis of BREEZE-AD1, AD2, and AD7 focused on skin pain and quality of life improvement in patients treated with baricitinib. Authors reported that a greater proportion of patients treated with baricitinib reached at least a 4-point reduction in Skin Pain NRS score at week 16 in BREEZE-AD1 (baricitinib 4 mg 25.3%, *p* < 0.001), -AD2 (baricitinib 4 mg 20.0%, *p* < 0.001; baricitinib 2 mg 19.0%, *p* < 0.001), and -AD7 (baricitinib 4 mg 48.8%, *p* < 0.001; baricitinib 2 mg 45.2%, *p* = 0.004) compared to placebo. A significantly higher proportion of Skin Pain NRS responders also achieved at least a 4-point improvement in DLQI at week 16 when compared with Skin Pain NRS non-responders in BREEZE-AD1 (89.2%, *p* < 0.0001), -AD2 (92.5%, *p* < 0.0001), and -AD7 (88.3%, *p* < 0.0001). [40]. Finally, considering a post-hoc analysis conducted by Buhl et al. about results from BREEZE AD1, AD2, and AD7, a rapid onset of action, typically one day after taking the first dose of baricitinib, was observed consistently for the burdensome symptoms of itch and sleep disturbance [41]. Long-term results on the efficacy of baricitinib for the treatment of AD are expected from BREEZE-AD3, AD4, and AD5 long-term extension. Finally, another phase III multicenter, randomized, double-blind, placebo-controlled, parallel-group, outpatient trial evaluating the pharmacokinetics, efficacy, and safety of baricitinib in pediatric patients with moderate to severe atopic dermatitis is now ongoing. Children aged between 2 years to 17 years who have had inadequate response or intolerance to existing topical medications are currently being recruited from several global countries [33].

### 3.3. Safety

From data available about safety, treatment-emergent Adverse Events (TEAEs) were reported in 54, 54, 58 and 58% of patients and in 56, 53, 58, and 54% of patients on placebo and baricitinib 1, 2, and 4 mg in BREEZE-AD1 and BREEZE-AD2, respectively. Nasopharyngitis, upper respiratory tract infections (URIs), creatine phosphokinase (CPK) elevations, and headaches were the most frequently reported AEs (>2% in any treatment group). Headaches were mild (76% of reported cases) and short-lived (median 0–5 days), with none requiring study-drug interruption or discontinuation. CPK elevations were asymptomatic and either resolved to below the upper limit of normal or were solved during the study without treatment interruption. According to the Common Terminology Criteria for Adverse Events (CTCAE), 16 of 20 cases were grade 3 or higher on baricitinib groups: three patients needed a temporary drug interruption, while one discontinued the study. In detail, grade 3 or 4 CPK elevations were seen in 1.6, 2.4, 3.3, and 2.4% of patients and in 2.1, 1.7, 1.7, and 4.9% of patients on placebo and baricitinib 1, 2, and 4 mg in BREEZE-AD1 and BREEZE-AD2, respectively. During the treatment period, 15 (3%) serious adverse events (SAEs) were reported with placebo vs. 10 (4%), 3 (1.2%), and 3 (1.2%) in the baricitinib 1-, 2-, and 4-mg groups, respectively. No cardiovascular events, venous thromboembolism, gastrointestinal perforation, significant hematological changes, or death were observed with any baricitinib dosage. No clinically meaningful changes in haemoglobin, neutrophil, lymphocyte, and platelet counts were observed in the study groups [35].

In BREEZE-AD7, among the 329 patients enrolled, the overall percentage of TEAEs was higher in the 4-mg (57.7%) and in the 2-mg baricitinib (56%) groups than placebo (38%). There was a greater proportion of patients with one or more treatment-emergent infections in the 4-mg baricitinib group (37 of 111 (33%)) and 2-mg baricitinib group (41 of 109 (38%)) than in the placebo group (26 of 108 (24%)). The most frequently reported (≥2% in any treatment group) TEAEs for both baricitinib doses compared with placebo were nasopharyngitis, folliculitis, oral herpes, upper respiratory tract infection, acne, diarrhea, and back pain. Some hematologic changes were reported during the trial in the treatment groups: neutrophil and lymphocyte count changes (3 cases (3% of baricitinib 4 mg + TCS), platelet count increases (2 cases (2% of baricitinib 2 mg + TCS), high-density lipoprotein level and low-density lipoprotein level increases (40 cases (20% of the 2 mg + TCS group and 31% of the 4 mg + TCS group), and alanine aminotransferase level increases greater than or equal to 3 times the upper limit of normal in the study. Several cases of creatinine phosphokinases elevation occurred according the CTCAE as grade 1 (14 and 23%) or grade 2 (7 and 6%) and few grade 3 (3 and 4%) and grade 4 (1 and 1%) of the 2- and 4-mg + TCS arms, respectively. Serious adverse events were reported in four patients (4%) in the 4-mg + TCS group, two (2%) in the 2-mg + TCS group, and four (4%) in the placebo + TCS group. No malignant tumors, major adverse cardiovascular events (MACE), or deaths were reported [36].

The safety results of BREEZE-AD5 after 16 weeks of treatment at baricitinib 2 mg were similar to previous phase II studies in terms of TEAEs and SAE frequency [37]. Main safety results available from clinicaltrials.gov are summarized in Table 3.

King et al. conducted an extended safety analysis of baricitinib 2 mg by integrating results from phase II and phase III RCTs. They divided the study population in two datasets: the first dataset compared the safety of baricitinib 2 mg with placebo in six 16-week studies, and TEAEs were higher for baricitinib 2 mg (57.9%) vs. placebo (51.6%), while serious adverse events, serious infections, and opportunistic infections were low in frequency and similar between baricitinib 2 mg and placebo; results confirmed the absence of occurrences of cancer, gastrointestinal perforations, or major adverse cardiovascular events within the first 16 week of treatment. The second dataset included an additional two extension studies and examined the safety of baricitinib in all patients receiving at least one dose of baricitinib 2 mg: 1598 patients received once-daily baricitinib 2 mg for 1434.2 patient-years of exposure, for a maximum of 2.4 years, with a median time of 330 days. There were five reports of cancer other than non-melanoma skin cancer, two major adverse cardiovascular events, one peripheral venous thrombosis, one arterial thrombosis, and no pulmonary embolisms, deep vein thrombosis, or deaths [42]. Pooled safety analyses have been conducted by Bieber et al. [43], including 4-mg-treated arms in addition to the analysis of King et al. Data were collected for 2531 patients who were given baricitinib for 2247 patient-years (median duration 310 days). After 16 weeks of treatment, during the placebo-controlled period, frequency of TEAEs was slightly higher in the baricitinib treatment groups compared to placebo. No malignancies, gastrointestinal perforations, cardiovascular events, or tuberculosis were observed. Authors noticed in long-term extension results of baricitinib 4-mg arms two venous thrombosis events and one death and higher frequency of herpes simplex in the 4-mg group (6.1%) vs. the 2-mg (3.6%) and placebo group (2.7%); the most common serious infections were eczema herpeticum (*n* = 11, incidence rates (IR) = 0.5), but the overall incidence of serious infection was low and similar between treated patients and placebo arms. In the long-term extensions (LTEs), two major adverse cardiovascular events in baricitinib 2 mg, two venous thrombosis events in baricitinib 4 mg, and one death occurred [43].

## 4. Discussion

Atopic dermatitis is the most prevalent chronic inflammatory skin condition in the word. AD affects children and adults with significant impact on the lives of patients, caregivers, and family members [5,6,44]. The burden of atopic dermatitis is extensive and reflects on quality of sleep, work productivity, emotional and mental health, physical activity, and social functioning of patients suffering from AD [45]. For these reasons, the growing interest in understanding the pathogenetic mechanisms of the disease and the identification of the clinical phenotypes has led to develop new therapeutic strategies.

### 4.1. Therapeutic Uses of JAK Inhibitors

The JAK inhibitors have long been used in the treatment of other immune-mediated diseases, such as rheumatoid arthritis [46], or hematologic disorders, such as mielofibrosis [47]. Janus kinasis, by the Jak/STAT signaling, plays an important role in the immune and hematopoietic function, but it is also involved in the pathogenetic pathway of inflammatory diseases. Baricitinib has also been considered by the EMA as a treatment against COVID-19 in hospitalized patients from 10 years of age who require supplemental oxygen. The inhibitory action on “the cytokine storm” seen in severe SARS-CoV-2 has been demonstrated by a double-blind, randomized, placebo-controlled trial evaluating baricitinib plus remdesivir in hospitalized adults with COVID-19. Baricitinib plus remdesivir was superior to remdesivir alone in reducing recovery time and accelerating improvement in clinical status among patients with COVID-19. The combination was associated with fewer serious adverse events [48]. Finally, baricitinib is currently under development to gain indication as treatment for alopecia areata in adult patients [49,50].

### 4.2. Baricitinib: Evidence in Atopic Dermatitis

Several JAK inhibitors are under study for the approval on the treatment of atopic dermatitis, and baricitinib is the first to be approved [14]. One phase II trial [25] and seven phase III trials belonging to BREEZE-AD program [26,27,28,29,30,31,32] evaluated the efficacy and safety of baricitinib in adult subjects suffering from moderate to severe AD. Another phase III study is ongoing on pediatric population [33].

#### 4.2.1. Efficacy on Eczema

Currently, data available have shown the superiority of baricitinib in monotherapy (BREEZE-AD1, AD2, and AD5) or in association with TCS (Phase II, BREEZE-AD7, AD4) compared to placebo at week 16 of treatment. Baricitinib was administered once daily, orally, and the different therapeutic dosages have been tested in double blind. The results showed a statistically significative difference between 4-mg therapeutic dosage regime and placebo in terms of efficacy, measured by two main primary endpoints among all of these trials: the percentage of patients achieving an IGA score 0–1 (with a 2-point or greater improvement from baseline) or an improvement of 75% of EASI score from baseline (EASI 75) [34,35,36,37]. The 2-mg dose was not as effective as the 4 mg but still more effective than placebo, with statistically significant differences observed in the studies in which baricitinib was administered in monotherapy (BREEZE-AD1, AD2, and AD5) at week 16 [35,37].

#### 4.2.2. Efficacy on Symptoms and Quality of Life

Pruritus, a major AD symptom, as well as impact of itch on sleep, showed significant and rapid improvement with baricitinib 4 mg as early as week 1 and continued to show improvement through week 16, with a consequent quality of life improvement of patients [38,39,40,41].

#### 4.2.3. Safety and Tolerability

As regards safety baricitinib showed a good safety profile after 16 weeks of treatment. Previously, safety results from baricitinib in the treatment of AR [46] had raised the alert level for the occurrence serious of adverse events (malignancies, thrombosis, and serious infections) that did not occur in studies for AD, maybe due to the different disease-specific characteristics of study populations more than to the treatment with baricitinib. No malignant tumors, major adverse cardiovascular events, thromboembolism, or deaths were reported in phase II and phase III trials for AD in the first 16 weeks of treatment. The most frequently reported TEAEs were mild infections in details nasopharyngitis, upper respiratory tract infections, and oral herpes. Herpes simplex was observed more often with baricitinib only in BREEZE-AD1. Increases of CPK have been reported, most of them of no clinical relevance. The frequency of SAE was low and similar to placebo. In the LTEs pooled data, one death, two cases of MACE, and two venous thrombosis occurred, highlighting the good safety profile [42,43].

### 4.3. Regulatory Affair

Baricitinib is the first JAK inhibitor (selective and reversible inhibitor of JAK 1 and JAK2) that has been approved in Europe by EMA on 17 September 2020 for the treatment of moderate to severe AD [24]. Baricitinib is actually approved in 40 countries, including Japan and Italy: in Italy, it is approved but not reimbursable, waiting for additional comparison data vs. dupilumab requested by the AIFA Price and Reimbursement Committee. The list of European countries in which baricitinib is already reimbursed in treating AD is reported in Table 4. In other countries, like the United States (U.S.), baricitinib is under investigation for the treatment of moderate to severe AD in adult patients who are candidates for systemic therapy. In a poster presented at Virtual Ispor on May 2021 [51], the authors presented the budget impact of baricitinib in the U.S. for moderate to severe psoriasis, and they concluded that the addition of baricitinib to a formulary budget would result in a cost-saving budget impact across all disease severities (mild, moderate, and severe), with the greatest relative cost-savings in the moderate AD population, where baricitinib market uptake is expected to be the highest. Additionally, considering the annual monitoring cost of a therapy with JAK inhibitors, baricitinib is expected to be a cost-saving addition to a plan’s budget based on its lower annual cost compared to dupilumab. In Europe, according to guidance from the National Institute for health and Care Excellence (NICE), annual treatment for patients with baricitinib will be less expensive than annual treatment with dupilumab, with an estimated price of about less than half the cost of dupilumab [52]. However, the results of the indirect treatment comparison suggest that baricitinib is less effective than dupilumab. Further budget impact analysis in Europe is needed, considering the prevalence and severity of the disease and the related direct and indirect costs given the available treatment alternatives. According NICE guidance, baricitinib and dupilumab are likely to be used in a sequence, but the reliability of sequencing analyses is uncertain. Baricitinib would be used after at least one systemic immunosuppressant; a composite end point of EASI 50 plus an improvement in the DLQI of at least 4 is most relevant for decision making [52].

### 4.4. Posology and Precautions for Use

According to efficacy and safety results, the recommended dose of baricitinb in the treatment of AD is 4 mg once a day. A dose of 2 mg once daily is appropriate for patients ≥75 years of age and may be appropriate for patients with a history of chronic or recurrent infections. A dose of 2 mg once daily should be considered for patients who have achieved persistent control of disease activity with a dose of 4 mg once daily and are eligible for a dose reduction. The efficacy of baricitinib may be enhanced when administered with topical corticosteroids and the use of topical calcineurin inhibitors is allowed but should be limited to sensitive areas only, such as the face, neck, folds, and genitals. Here are some recommendations from the technical data sheet before the use of baricitinib in AD [53]: patients should be screened for tuberculosis (TB) before starting therapy with baricitinib, which must not be given to patients with active tuberculosis. Therapy anti-TB should be considered before starting baricitinib therapy in non-patients previously treated with latent TB. Screening for viral hepatitis should be done in accordance with clinical guidelines prior to initiate therapy. Data from clinical trials are available about patients who tested positive for hepatitis C antibodies but negative for hepatitis C virus RNA and patients with surface antibodies and core hepatitis B antibodies, without hepatitis B surface antigen, who were allowed to receive the treatment [53].

### 4.5. Upcoming Prospects

Further data on long-term efficacy and safety of baricitinib and real-life studies are needed to better define the position of baricitinib in the therapeutic management of AD and to evaluate the maintenance of therapeutic effects for the treatment of moderate to severe AD in adults by the time. First data from pediatric trial will be available on 2022. The advantages of baricitinib in the treatment of AD are linked to the rapidity of action especially on symptoms, in particular itch, which is associated with an equally rapid improvement in the patient’s quality of life; the oral route of administration may be another strength compared to dupilumab, currently the only alternative to traditional systemic therapies, administered by injection [54,55]. Other competitors belonging to JAK inhibitors family (such as upadacitinib or abrocitinib) and monoclonal antibodies (anti IL-13 and anti IL-31) will be soon available. Head to head studies will be useful to compare efficacy and safety profile of these molecules to define the therapeutic algorithm of AD considering patients’ age, the severity of the disease, and clinical phenotypes. Just as the growing knowledge of the multiple aspects of the pathogenesis of AD led to recognize new target molecules, future data could lead to a clinical laboratory correlation with the identification of specific biomarkers disease. The experimental program of baricitinb involved the identification of biomarkers for the evaluation of the therapeutic response, which are difficult to identify as per clinical practice. In particular, from AD studies the serum levels of IL-19, a pro-proliferative marker of keratinocytes, during treatment with baricitinib resulted to be closely related to EASI improvement [55,56].

## 5. Conclusions

Baricitinib, a selective JAK1 and JAK 2 inhibitor, is a new oral small molecule now available in Europe for the treatment of moderate to severe AD in adult patients. Efficacy and safety results from pivotal trials confirmed the superiority of 4-mg dosage regime compared to placebo at week 16, even in association with TCS, with a good safety profile. The main advantage of baricitinib is the rapid onset of improvement in symptoms, especially pruritus, from the first days of treatment. Further data are needed to evaluate long-term efficacy and safety in a real-life setting.

## Figures and Tables

**Table 1:** Main characteristics of the BREEZE-AD program phase III studies.

**BREEZE-AD1** NCT0334396 I4V-MC-JAHL	GLOBAL ***n* = 660** **monotherapy**	A Multicenter, Randomized, Double-Blind, Placebo-Controlled, Phase 3 Study to Evaluate the Efficacy and Safety of Baricitinib in Adult Patients With Moderate to Severe Atopic Dermatitis	Study Duration **W16** oral 4 mg:2 mg:1 mg:PBO	PRIMARY ENDPOINT IGA 0–1 W16	Completed
**BREEZE-AD2** NCT03334422 I4V-MC-JAHM	GLOBAL ***n* = 615** **monotherapy**	A Multicenter, Randomized, Double-Blind, Placebo-Controlled, Phase 3 Study to Evaluate the Efficacy and Safety of Baricitinib in Patients With Moderate to Severe Atopic Dermatitis	Study Duration **W16** oral 4 mg:2 mg:1 mg:PBO	PRIMARY ENDPOINT IGA 0–1 W16	Completed
**BREEZE-AD3** NCT03334435; I4V-MC-JAHN	GLOBAL ***n* = 1760** **(allowed TCS)**	A Phase 3, Multicenter, Double-Blind Study to Evaluate the Long-Term Safety and Efficacy of Baricitinib in Adult Patients With Atopic Dermatitis	Study Duration **W104** oral High:mild:low:PBO	PRIMARY ENDPOINT IGA 0–1 W16,36,52	Active, not recruiting
**BREEZE-AD4** NCT03428100 I4V-MC-JAIN	GLOBAL ***n* = 463** **(TCS combo)**	A Phase 3, Multicenter, Double-Blind, Randomized, Placebo-Controlled Study Evaluating the Safety and Efficacy of Baricitinib in Combination With Topical Corticosteroids in Adult Patients With Moderate to Severe Atopic Dermatitis Who Have Experienced Failure to Cyclosporine or Are Intolerant to or Have Contraindication to Cyclosporine	Study Duration **W104** oral 4 mg:2 mg:1 mg:PBO	PRIMARY ENDPOINT EASI 75 W16	Active, not recruiting
**BREEZE-AD5** NCT03435081; I4V-MC-JAIW	US/CANADA ***n* = 440** **monotherapy**	A Multicenter, Randomized, Double-Blind, Placebo-Controlled, Phase 3 Study to Evaluate the Efficacy and Safety of Baricitinib in Adult Patients With Moderate to Severe Atopic Dermatitis	Study Duration **W104** oral 2 mg:1 mg:PBO	PRIMARY ENDPOINT EASI 75 W16	Completed
**BREEZE-AD6** NCT03559270; I4V-MC-JAIX	US/CANADA ***n* = 380** **(allowed TCS)**	A Multicenter, Open-Label, Phase 3 Study to Evaluate the Efficacy and Safety of Baricitinib in Adult Patients With Moderate to Severe Atopic Dermatitis	Study Duration **W104** oral 2 mg	PRIMARY ENDPOINT EASI 75 W16	Active, not recruiting
**BREEZE-AD7** NCT0373301; I4V-MC-JAIY	GLOBAL ***n* = 329** **(TCS combo)**	A Multicenter, Randomized, Double-Blind, Placebo-Controlled, Phase 3 Study to Evaluate the Efficacy and Safety of Baricitinib in Combination With Topical Corticosteroids in Adult Patients With Moderate to Severe Atopic Dermatitis BREEZE-AD7	Study Duration **W16** oral 4 mg:2 mg:PBO	PRIMARY ENDPOINT IGA 0–1 W16	Completed

Abbreviations: PBO, placebo; TCS, topical corticosteroid; IGA, investigator global assessment; EASI, Eczema Area Severity Index.

**Table 2:** Summary of sources cited.

Authors [Ref]	Title	Type of Study	Source of Data	Information about Publication
Promoted by Eli Lilly and Company [25]	A Study of Baricitinib (LY3009104) in Participants With Moderate to Severe Atopic Dermatitis	Phase II RCT	clinicaltrials.gov NCT02576938 (accessed on 2 November 2021)	Last update posted 17 June 2020
Promoted by Eli Lilly and Company [26]	A Study of Baricitinib (LY3009104) in Patients With Moderate to Severe Atopic Dermatitis (BREEZE-AD1)	Phase III RCT	clinicaltrials.gov NCT03334396 (accessed on 2 November 2021)	Last update posted 18 August 2020
Promoted by Eli Lilly and Company [27]	Study of Baricitinib (LY3009104) in Patients With Moderate to Severe Atopic Dermatitis (BREEZE-AD2)	Phase III RCT	clinicaltrials.gov NCT03334422 (accessed on 2 November 2021)	Last update posted 22 January 2020
Promoted by Eli Lilly and Company [28]	A Study of Long-term Baricitinib (LY3009104) Therapy in Atopic Dermatitis (BREEZE-AD3)	Phase III RCT	clinicaltrials.gov NCT03334435 (accessed on 2 November 2021)	No results posted
Promoted by Eli Lilly and Company [29]	A Long-term Study of Baricitinib (LY3009104) With Topical Corticosteroids in Adults With Moderate to Severe Atopic Dermatitis That Are Not Controlled With Cyclosporine or for Those Who Cannot Take Oral Cyclosporine Because it is Not Medically Advisable (BREEZE-AD4)	Phase III RCT	clinicaltrials.gov NCT03428100 (accessed on 2 November 2021)	Last update posted 11 May 2021 (pre-print)
Promoted by Eli Lilly and Company [30]	A Study of Baricitinib (LY3009104) in Adult Participants With Moderate to Severe Atopic Dermatitis (BREEZE-AD5)	Phase III RCT	clinicaltrials.gov NCT03435081 (accessed on 2 November 2021)	Last update posted 19 August 2021
Promoted by Eli Lilly and Company [31]	A Study of Baricitinib (LY3009104) in Participants With Moderate to Severe Atopic Dermatitis (BREEZE-AD6)	Phase III RCT	clinicaltrials.gov NCT03559270 (accessed on 2 November 2021)	No results posted
Promoted by Eli Lilly and Company [32]	A Study of Baricitinib (LY3009104) in Combination With Topical Corticosteroids in Adults With Moderate to Severe Atopic Dermatitis (BREEZE-AD7)	Phase III RCT	clinicaltrials.gov NCT03733301 (accessed on 2 November 2021)	Last update posted 11 August 2020
Promoted by Eli Lilly and Company [33]	A Study of Baricitinib (LY3009104) in Children and Adolescents With Atopic Dermatitis (BREEZE-AD-PEDS)	Phase III RCT	clinicaltrials.gov NCT03952559 (accessed on 2 November 2021)	No results posted
Guttman-Yassky et al. [34]	Baricitinib in adult patients with moderate to severe atopic dermatitis: a phase 2 parallel, double-blinded, randomized placebo-controlled multiple-dose study.	PHASE II RCT	NCT02576938	Published on 2019 J Am Acad Dermatol.
Simpson EL et al. [35]	Baricitinib in patients with moderate to severe atopic dermatitis and inadequate response to topical corticosteroids: results from two randomized monotherapy phase III trials.	Phase III RCT	**BREEZE-AD1** NCT03334396 **BREEZE-AD2** NCT03334422	Published on August 2020 Br J Dermatol.
Reich K. et al. [36]	Efficacy and Safety of Baricitinib Combined With Topical Corticosteroids for Treatment of Moderate to Severe Atopic Dermatitis: A Randomized Clinical Trial.	Phase III RCT	**BREEZE-AD7** NCT03733301	Published on December 2020 JAMA Dermatol
Simpson EL et al. [37]	Baricitinib in patients with moderate-to-severe atopic dermatitis: Results from a randomized monotherapy phase 3 trial in the United States and Canada (BREEZE-AD5)	Phase III RCT	**BREEZE-AD5** NCT03435081	Published on July 2021 J Am Acad Dermatol.
Reich K. et al. [38]	Baricitinib improves symptoms in patients with moderate to severe atopic dermatitis and inadequate response to topical corticosteroids: patient-reported outcomes from two randomized monotherapy phase III trials	Phase III RCT	**BREEZE-AD1** NCT03334396 **BREEZE-AD2** NCT03334422	Published on November 2020 J Dermatolog Treat Epub ahead of print
Wollemberg A. et al. [39]	Impact of baricitinib in combination with topical steroids on atopic dermatitis symptoms, quality of life, and functioning in adult patients with moderate to severe atopic dermatitis from the BREEZE-AD7 Phase 3 randomised trial	Phase III RCT	**BREEZE-AD7** NCT03733301	Published on July 2021 J Eur Acad Dermatol Venereol.
Thyssen JP et al. [40]	Baricitinib Rapidly Improves Skin Pain Resulting in Improved Quality of Life for Patients with Atopic Dermatitis: Analyses from BREEZE-AD1, 2, and 7	Phase III RCTs	**BREEZE-AD1** NCT03334396 **BREEZE-AD2** NCT03334422 **BREEZE-AD7** NCT03733301	Published on October 2021 Dermatol Ther
Buhl T. [41]	Itch and Sleep Improvements with Baricitinib in Patients with Atopic Dermatitis: A Post-Hoc Analysis of 3 Phase 3 Studies.	Phase III RCTs	**BREEZE-AD1** NCT03334396 **BREEZE-AD2** NCT03334422 **BREEZE-AD7** NCT03733301	Published on June 2021 Dermatol Ther
King B. et al. [42]	Extended Safety Analysis of Baricitinib 2 mg in Adult Patients with Atopic Dermatitis: An Integrated Analysis from Eight Randomized Clinical Trials	Phase II–III RCTs	NCT02576938 **BREEZE-AD1** NCT03334396 **BREEZE-AD 2** NCT03334422 **BREEZE-AD3** NCT03334435 **BREEZE-AD4** NCT03428100 **BREEZE-AD5** NCT03435081 **BREEZE-AD6** NCT03559270 **BREEZE-AD7** NCT03733301	Published on May 2021 Am J Clin Dermatol
Bieber T. et al. [43]	Pooled safety analysis of baricitinib in adult patients with atopic dermatitis from 8 randomized clinical trials.	Phase III RCTs	**BREEZE-AD1** NCT03334396 **BREEZE-AD2** NCT03334422 **BREEZE-AD3** NCT03334435 **BREEZE-AD4** NCT03428100 **BREEZE-AD5** NCT03435081 **BREEZE-AD6** NCT03559270 **BREEZE-AD7** NCT03733301	Published on February 2021 J Eur Acad Dermatol Venereol.

Abbreviations: RCT, randomized controlled trials; ref: references.

**Table 3:** Summary of adverse events from BREEZE-AD1, AD2, AD4, AD5, and AD7 phase III studies currently available on clinicaltrials.gov. (accessed on 2 November 2021).

	BREEZE-AD1			BREEZE-AD2			BREEZE-AD4			BREEZE-AD5		BREEZE-AD7		
Number of Patients	125	123	249	123	123	244	92	184	93	145	146	111	109	108
Baricitinib dosage	4 mg	2 mg	PBO	4 mg	2 mg	PBO	4 mg + TCS	2 mg + TCS	PBO + TCS	2 mg	PBO	4 mg + TCS	2 mg + TCS	PBO + TCS
TEAES	73 (58.4%)	71 (57.7%)	135 (54.2%)	66 (53.7%)	71 (57.7%)	137 (56%)	50 (54.3%)	69 (37.5%)	29 (31.2%)	18 (12.4%)	20 (13.7%)	64 (57.7%)	61 (56%)	41 (38%)
Nasopharingitis	12 (9.6%)	12 (9.8%)	26 (10.4%)	10 (8.1%)	16 (13%)	30 (12%)	27 (29.3%)	34 (18.5%)	13 (14%)	9 (6.1%)	11 (7.5%)	17 (15%)	12(11%)	13 (12%)
URIs	4 (3.2%)	3 (2.4%)	6 (2.4%)	4 (3.3%)	5 (4.1%)	5 (2%)	10 (11%)	10 (5.4%)	2 (2.1%)	11 (7.6%)	9 (6.2%)	3 (3%)	8 (7%)	2 (2%)
Herpes simplex	9 (7.2%)	4 (3.3%)	3 (1.2%)	5 (4.1%)	7 (5.7%)	11 (4.5%)	5 (5.4%)	5 (2.7%)	4 (4.3%)	nr	nr	3 (3%)	1 (1%)	3 (3%)
Headache	10 (8%)	14 (11.4%)	16 (6.4%)	11 (8.9%)	9 (7.3%)	5 (2%)	9 (9.8%)	11 (6%)	6 (6.5%)	nr	nr	nr	nr	nr
CPK elevations	4 (3.2%)	1 (0.8%)	2 (0.8%)	7 (5.7%)	1 (0.8%)	1 (0.4%)	nr	nr	nr	nr	nr	0	3 (3%)	0
SAE	2 (1.6%)	0	6 (2.4%)	1 (0.8%)	3 (2.4%)	9 (3.7%)	6 (6.5%)	4 (2.2%)	2 (2.2%)	2 (1.4%)	3 (2%)	4 (4%)	2 (2%)	4 (4%)
MACE	0	0	0	0	0	0	0	2	0	0	0	0	0	0
Death	0	0	0	0	0	0	0	0	0	0	0	0	0	0
Malignant	0	0	0	0	0	0	0	0	0	0	0	0	0	0

Results refer to W16. Abbreviations; PBO, placebo; TCS, topical corticosteroid; TEAE, treatment emergent adverse event; URIs, upper respiratory infections; CPK, creatine phosphokinase; SAE, serious adverse event; MACE, major adverse cardiovascular events; nr, not reported.

**Table 4:** List of European countries in which baricitinib is already reimbursed in treating atopic dermatitis.

Baricitinib Reimbursed EU Contries for Atopic Dermatitis	
Austria	Norway
Denmark	Sweden
Finland	Switzerland
France	UK
Germany	Scotland
Netherlands	Slovenia
Portugal	Spain (end 2021)

## Data Availability

https://clinicaltrials.gov/ct2/show/results/NCT03334396, (accessed on 2 November 2021). NCT03334422, NCT03334435, NCT03428100, NCT03435081, NCT03559270, NCT03733301, NCT03952559.
